# Overweight/Obese Schoolchildren with Low Muscle Strength Have a Lower Cardiorespiratory Capacity and Greater Cardiovascular Risk: Results of the School Health Survey of the Extreme South of Chile 2019

**DOI:** 10.3390/children8090734

**Published:** 2021-08-26

**Authors:** Javier Albornoz-Guerrero, Rafael Zapata-Lamana, Daniel Reyes-Molina, Igor Cigarroa, Guillermo García Pérez de Sevilla, Sonia García-Merino

**Affiliations:** 1 Departamento de Educación y Humanidades, Universidad de Magallanes, Punta Arenas 6200000, Chile; Javier.albornoz@umag.cl; 2 Escuela de Educación, Universidad de Concepción, Los Ángeles 4440000, Chile; rafaelzapata@udec.cl; 3 Facultad de Ciencias Sociales, Universidad de Concepción, Concepción 4030000, Chile; danielreyes@udec.cl; 4 Escuela de Kinesiología, Facultad de Salud, Universidad Santo Tomás, Santiago 8320000, Chile; icigarroa@santotomas.cl; 5 Faculty of Sports Sciences, Department of Physiotherapy, Universidad Europea de Madrid, 28670 Madrid, Spain; guillermo.garcia@universidadeuropea.es; 6 Sports Sciences Department, Faculty of Health Sciences, Francisco de Vitoria University, 28223 Madrid, Spain

**Keywords:** exercise, cardiorespiratory fitness, strength, obesity, children

## Abstract

Objective: To compare cardiovascular risk and cardiorespiratory capacity in schoolchildren from a region in the extreme south of Chile according to nutritional status and muscular strength. Methods: An analytical cross-sectional study was performed on a sample of 594 schoolchildren from 5th to 8th grade in the extreme south of Chile. Based on body mass index and lower limb muscle strength, participants were divided into four groups: high strength-normal weight, high strength-overweight/obese, low strength-normal weight, and low strength-overweight/obese. Then, waist-to-height ratio and cardiorespiratory capacity, measured with the 20 m shuttle run test, were assessed to determine their cardiovascular risk, comparing the four groups. Results: The overweight/obese group with high muscular strength presented better indicators in anthropometric variables (waist circumference and waist-to-height ratio) than their peers with low muscular strength. Additionally, the overweight/obese group with low muscular strength presented a lower cardiorespiratory capacity than their peers with high muscular strength. Both results were observed in boys and girls. Conclusion: The results of this study suggest that overweight/obese schoolchildren with high muscle strength present healthier anthropometric indicators and greater cardiorespiratory capacity than their peers with low muscle strength. These results confirm the relevance of measuring muscle strength in schoolchildren and its usefulness to assess functionality. These results encourage the scientific community to continue studying the role that muscle strength plays in modulating the effects of overweight and obesity on respiratory and cardiovascular conditions in childhood.

## 1. Introduction

Childhood obesity is one of the principal public health problems in the world [1,2], with more than 340 million children and adolescents being overweight or obese in 2016 [3]. In this sense, children with excessive adiposity present a higher cardiovascular risk during childhood and adolescence [4,5,6,7,8] and then premature mortality when becoming adults [9,10]. If a low physical fitness is added to overweight/obesity, cardiovascular risk factors will significantly increase [11,12].

Physical fitness plays a preventive role against numerous diseases [11,13]. In this regard, the main components of physical fitness are cardiorespiratory capacity, which allows supplying oxygen during sustained physical activity; muscle strength; flexibility, which is the ability of the muscles to move freely through a full joint range of motion; and motor ability, which includes speed, agility, and balance [14].

Along these lines, there is ample evidence on how physical fitness, and particularly cardiorespiratory capacity and muscular strength, are associated with a lower rate of cardiovascular risk factors, and with numerous health benefits for children. [15,16,17,18,19]. Thus, moderate to high cardiorespiratory capacity levels in this population can attenuate the metabolic consequences of mortality attributed to an excess of adiposity [12,20,21]. Therefore, cardiorespiratory capacity could be an essential variable to consider and promote in children regardless of their obesity levels [22].

During the last decade, there has been growing evidence about the “fat but fit” paradox, in which overweight people with good physical fitness have better health indicators and lower cardiovascular risk than people with a healthy weight and low physical fitness [2]. However, the evidence regarding the relationship between muscle strength and cardiorespiratory capacity in children with different nutritional statuses is still not clear.

There is a negative relationship between muscle strength and anthropometric variables, such as body mass index (BMI) and waist circumference [17,23]. Muscle strength should be analyzed when assessing nutritional status and body composition abnormalities; a positive relationship between high muscle strength and lower cardiovascular risk has also been mentioned in children, especially when accompanied by an ideal weight [17,22,24,25,26,27]. Therefore, muscle strength can be considered a marker of general health in children.

It is necessary to evaluate these health markers in vulnerable children who are overweight/obese. In this sense, a recent systematic review indicates that overweight is more prevalent in countries with extreme cold weather [28]. In this type of climate, both physical inactivity [29,30] and the development of multiple systemic adaptations that lead to the development of obesity are favored [31]. For example, alterations in hormonal production, such as increased ghrelin and cortisol secretion, have been associated with an increased appetite and lipid storage mechanisms [32].

Among the coldest areas of the world is the extreme South of Argentina and Chile. Particularly in Punta Arenas, Chile, the temperature varies around an average of 6.5 °C, with the lowest temperature of −16.4 °C in winter and the highest of 29.9 °C in summer [33]. In line with the aforementioned, this locality has the highest levels of childhood obesity in Chile [34]. However, no data have currently been reported from areas with extreme cold weather, nor is there updated evidence on cardiovascular risk and physical fitness, specifically on cardiorespiratory capacity and muscle strength in school children from the extreme south of Chile.

Therefore, the objective of this study was to compare cardiovascular risk and cardiorespiratory capacity in schoolchildren from the extreme South of Chile according to nutritional status and muscular strength.

## 2. Methodology

Study design: An observational, analytical, cross-sectional study was carried out.

Participants: Schoolchildren from 5th to 8th grade from 3 of the 19 urban public educational establishments in the Magallanes and Chilean Antarctic regions participated. The sample size of 615 children was obtained considering 50% of heterogeneity, a margin of error of 5%, and a confidence level of 95%. Of those 615 children, 14 were excluded for not signing the informed consent, 4 did not participate due to inability to perform the physical tests, and 3 were excluded because they did not complete all the evaluations, so the final sample was 594 schoolchildren.

The research team and the Municipal Corporation of the mentioned regions of Chile signed a collaboration agreement. Then, three schools were randomly selected. The study design and its planning were decided in conjunction with the management team and teachers of the educational establishments. Subsequently, a kinesiologist, a nutritionist, a psychologist, and a physical education teacher were recruited. They conducted a brief training session on assessment instruments to reduce the risk of inter-rater bias. Data collection was carried out at the end of the first semester of 2020, in adapted spaces within educational establishments, on the same day and at class time. The families, directors, teachers, and schoolchildren were informed about the purpose of the study and agreed to collaborate in it. All the schoolchildren who participated in the study gave their assent and their parents and legal guardians signed their signed consent. The project was approved by the Ethics Committee of the south-central macro zone of the Santo Tomás University, Chile, code number 96–20, and all the procedures were carried out in accordance with the Declaration of Helsinki and Singapore.

### 2.1. Variables

Variables to characterize the groups:

- Nutritional status: Bodyweight of the schoolchildren was measured with a SECA^®^ brand digital scale (model 804, Chino, CA, USA). The waist circumference was measured while standing with a 1.5 m tape, and the height was measured with a SECA^®^ brand portable stadiometer (model 213, Chino, CA, USA). These measures were performed according to the standardized procedures described by the International Society of Film anthropometry (ISAK) [35] and according to the Habicht method [36]. Subsequently, the BMI was calculated to obtain the BMI/age indicator, and to classify the nutritional status of each child according to sex, considering as malnutrition a standard deviation (SD) ≥ −2, risk of malnutrition SD ≥ −1, and normality between 0.99 and −0.99 SD. Overweight, obesity, and severe obesity were determined by values of ≥1 SD, ≥2 SD, and ≥3 SD, respectively [37].
- Muscle strength: The lower limbs’ muscle strength was evaluated through the long jump test, using the reference evaluations recommended by the Alpha Fitness battery for the school population in which low strength and high strength are defined according to age and sex. The mean for boys between 9 and 12.9 years old was 138.8 cm and the mean for girls between 9 and 12.9 years old was 121.6 cm [38].

Based on the nutritional status and the lower limbs’ muscle strength, participants were divided into four groups: high strength-normal weight, high strength -overweight/obese, low strength-normal weight, and low strength-overweight/obese.

Variables analyzed:

- Cardiovascular risk: For this, central obesity was analyzed, using the waist-to-height ratio, which allows categorizing participants into normal adiposity, moderate adiposity, and excessive adiposity [6,39,40,41,42].
- Cardiorespiratory capacity: The 20 m shuttle run test was used, measuring the time (s), the speed (m/s), and the number of shuttles completed, according to the protocol and reference values of the Alpha Fitness battery for the school population [38].

Additionally, sociodemographic values such as scholarship grade, place of residence, and whether children belonged to the school integration program were measured [43].

### 2.2. Statistical Analysis

Data were analyzed with the statistical software SPSS 25.0 (Windows, SPSS Inc., Chicago, IL, USA). Continuous variables were presented as mean and standard deviation, and categorical as percentages. After performing the Kolmogorov–Smirnov test to assess normality, all the variables showed a normal distribution, so parametric statistics were used. To establish an association between categorical variables, the chi-square test was used. To establish differences between the four groups (high strength-normal weight; high strength-overweight/obese; low strength-normal weight; low strength-overweight/obese), a one-factor ANOVA test (type of nutritional status and strength) was used, as well as a post hoc analysis (Bonferroni) to determine differences between the groups. The level of significance was set as *p* < 0.05.

## 3. Results

Table 1 shows the characteristics of the children included in the study. A higher number of boys were in the school integration program compared to girls (*p* = 0.003). In addition, a higher number of girls were in the high-strength category compared to boys (*p* = 0.005).

Table 2 shows the anthropometric characteristics of the total sample, according to group and sex. Significant differences were found between the groups in weight, BMI, waist circumference and waist-to-height ratio ((F(3.579) = 70.180; *p* = 0.000; F(3.579) = 162.489; *p* = 0.000; F(3.579) = 66.203; *p* = 0.000; F(3.579) = 79.302; *p* = 0.000, respectively)). Upon closer analysis, it was observed that the high strength-overweight/obese group had lower waist circumference (*p* = 0.000) and waist-to-height ratio (*p* = 0.000) than the overweight/obese low strength group. When segmenting the sample by sex, the same significant differences were evidenced between the groups in weight, BMI, waist circumference, and waist-to-height ratio in boys ((F(3.292) = 34.238; *p* = 0.000; F(3.292) = 85.568; *p* = 0.000; F(3.292)= 28.768; *p* = 0.000; F(3.292) = 32.097; *p* = 0.000, respectively)) and girls ((F(3.283) = 27.299; *p* = 0.000; F(3.283) = 78.328; *p* = 0.000; F(3.283) = 44.258; *p* = 0.000; F(3.283) = 50.058; *p* = 0.000, respectively)). Again, the high strength-normal weight and the low strength-normal weight groups had less weight, BMI, waist circumference, and waist-to-height ratio than the high strength-overweight/obese and low strength-overweight/obese groups. Additionally, specifically among girls, the high strength-overweight/obese group presented lower values of waist circumference (*p* = 0.000) and waist-to-height ratio (*p* = 0.000) than the low strength-overweight/obese group (Table 2).

**Figure 1:**
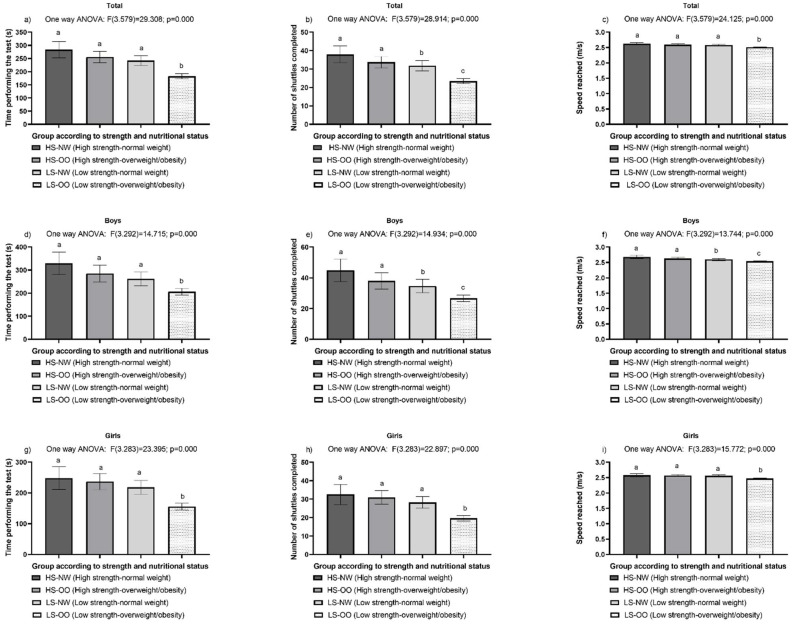
Characteristics of the cardiorespiratory capacity of the total sample, according to sex and group. HS, high strength; LS, low strength; NW, normal weight; OO, overweight/obese. Average a, b, c within a row with a different symbol indicates significant differences between groups (one-way ANOVA and post hoc comparison with Bonferroni test). A *p* < 0.05 was considered for all analyses.

Figure 1 shows the characteristics of the cardiorespiratory capacity of the total sample, according to group and sex. Significant differences between the groups in time, number of shuttles, and speed reached in the 20 m shuttle run test were evidenced in the total sample of schoolchildren ((F(3.592) = 29.308, *p* = 0.000; F(3.592) = 28.914, *p* = 0.000; F(3.592) = 24.125, *p* = 0.000, respectively)). When comparing groups, the low strength-overweight/obese group completed fewer shuttles and reached a lower speed than the rest of the groups. When comparing only the low strength groups, the overweight/obese children completed a fewer number of shuttles compared to the normal weight group (*p* = 0.000). When segmenting the sample by sex, the same significant differences were observed between the groups in time, the number of shuttles, and the speed reached in the 20 m shuttle run test in boys ((F(3.292) = 14.715, *p* = 0.000; F(3.292) = 14.934, *p* = 0.000; F(3.292) = 13.744, *p* = 0.000, respectively)) and girls ((F(3.283) = 23.395. *p* = 0.000; F(3.283) = 22.897, *p* = 0.000; F(3.283) = 11.772, *p* = 0.000, respectively)). In both boys and girls, again the low strength-overweight/obese group had the lowest performance in time, the number of shuttles completed, and speed reached in the test compared to the rest of the groups. When analyzing and comparing only the groups with low strength, the overweight/obese group also performed a lower number of shuttles (*p* = 0.000) and reached a lower speed (*p* = 0.000) than the normal weight group (Figure 1).

## 4. Discussion

The objective of this study was to analyze the relationship between health-related physical fitness and cardiovascular risk in schoolchildren from a region in the extreme South of Chile. The main findings suggest that the overweight/obese group with high muscular strength presented better indicators in anthropometric variables (waist circumference and waist-to-height ratio) than their peers with low muscular strength. Additionally, in the overweight/obese group, those with low muscle strength presented lower cardiorespiratory capacity than their peers with high muscular strength. Both results were observed in boys and girls.

### 4.1. Muscle Strength and Cardiovascular Risk

Concerning muscular strength and cardiovascular risk, the results of this study are in accordance with numerous authors, showing overweight children with high muscle strength have fewer cardio-metabolic risk factors than their peers who are overweight and have low muscular strength [17,22,24,25,26,44,45,46]. In this sense, these findings can be explained through the relationship between muscle strength and nutritional status. Along the same lines, various studies mention how BMI, waist circumference, and waist-to-height ratio are strong predictors of cardiovascular risk in children [4,5,6,7], as children with high adiposity tend to have an increased cardiovascular risk at the prepubertal stage [8].

Regarding muscle strength and anthropometric variables in children, an inverse relationship has been described between these variables, which can be explained by physiological and psycho-behavioral mechanisms [14,17,47]. In this regard, having high muscle strength contributes to enhancing daily energy expenditure, lipids oxidation, and glucose transport capacity. All this ultimately results in better anthropometric measures and nutritional status [48]. On the other hand, high levels of muscle strength encourage higher participation in physical activity [23], thus allowing better anthropometric and nutritional status indicators [49]. This may be because high muscle strength enhances the physical function [50], which increases self-efficacy of physical activity [51] and physical self-perception [52], which finally contributes to increasing physical activity levels [53,54].

Higher muscle strength and healthier body composition in childhood are associated with a healthier cardiovascular profile throughout life, in addition to lower all-cause mortality in adulthood [14,15,47,55]. Along the same lines, a recent prospective study, which measured lower limb muscle strength in children through the long jump test, indicated that a low level of muscle strength was associated with a poorer nutritional status, lower physical activity levels, and poorer cardiorespiratory fitness in adulthood [56].

Regarding muscle strength in childhood, Smith et al. (2019) pointed out that handgrip strength is a significant health indicator, but lower limb muscle strength is more clinically relevant. This is because the typical activities of children may not stimulate handgrip adaptations but rather displacement ones [57]. In this sense, a longitudinal study in children mentions that all the components of physical fitness were inversely associated with metabolic risk, except for upper body muscle strength [46]. In this regard, this study adds that metabolic risk in childhood could be modified by mainly improving cardiorespiratory capacity and lower body muscle strength [46].

### 4.2. Muscle Strength and Cardiovascular Capacity

Regarding cardiorespiratory capacity, the evidence shows a strong association between low cardiorespiratory capacity and increased cardiovascular risk factors in childhood [14,15]. In this sense, in our study, children with a healthy weight have a greater cardiorespiratory capacity, as reported in previous studies [48,58]. In addition, the results of this study could respond to “fat but strong”, which could be a new variant of the paradox of “fat but fit”. The overweight/obese group children with low muscle strength presented less cardiorespiratory capacity than their peers with high muscle strength. In this sense, studies have described positive associations between cardiorespiratory fitness and muscle strength in children [57,59,60].

In line with the findings mentioned above, a cross-sectional study that analyzed cardiorespiratory capacity and muscle strength in girls indicated that the overweight group had a lower cardiorespiratory capacity and muscle strength [61]. These lower muscle strength values could contribute to the lack of muscular power necessary to maintain a prolonged race, impacting the results in the cardiorespiratory capacity test [61]. This mechanism could explain the “fat but strong” paradox that arises in the present study.

Regarding cardiorespiratory capacity and muscle strength, the new guidelines of the World Health Organization suggest that children should perform strength exercises three days a week and cardiovascular exercises daily [62]. Concerning the benefits of physical activity in schoolchildren, the evidence indicates that cardiorespiratory capacity and muscular strength are positively associated with health-related quality of life [18], as well as with physical and psychological well-being [63].

Similarly, a study reported that cardiorespiratory capacity and muscular strength could counteract the adverse influence of being overweight on the academic performance of schoolchildren, where overweight children who presented greater cardiorespiratory capacity and muscle strength had better academic performance [64]. However, in the same way, weight control should be recommended to all obese people, regardless of their metabolic status, to reduce the risk of chronic diseases such as type 2 diabetes and respiratory diseases [65]. In addition, overweight children often present alterations associated with body dissatisfaction and health indicators, which limit their integral development in the preadolescent stage [58].

## 5. Strengths and Limitations

The cross-sectional nature of this study restricts the ability to determine any causality in the results. Furthermore, confounding variables that were not considered in the analysis, such as maturation stages, may have influenced the results. However, one of the strengths was the inclusion of different anthropometric measurement methods such as BMI, waist circumference, and waist-to-height index, which guarantees methodological quality and allows contrasting between nutritional status, cardiorespiratory capacity, and muscle strength. This study could encourage future research regarding cardiovascular risk factors and cardiorespiratory function, according to the nutritional status and muscle strength in children. However, for future studies, biomarkers that show stronger relationships between cardiovascular risk in overweight children and their physical fitness should be incorporated, especially for muscle strength.

## 6. Conclusions

This study suggests that overweight/obese schoolchildren with high muscular strength present better anthropometric indicators (waist circumference and waist-to- height ratio), and overweight/obese children with lower muscular strength present a lower cardiorespiratory capacity.

These results confirm the relevance of assessing muscle strength in schoolchildren and its usefulness as a marker of functionality. Additionally, this study could encourage the scientific community to continue studying the role that muscular strength plays as a modulator of the effects of excess nutrition on respiratory and cardiovascular conditions in childhood. In addition, these findings should be taken into account by health and education professionals who design and implement programs aimed at reducing the consequences of overweight and obesity on the physical and cardiovascular health of children.

## Figures and Tables

**Table 1:** Scholarship and health characteristics of the participants.

Grade	Boys	Girls	*p*
* Fifth grade*	73 (24.2%)	93 (31.8%)	0.079
* Sixth grade*	88 (29.1%)	65 (22.3%)	
* Seventh grade*	67 (22.2%)	56 (19.2%)	
* Eighth grade*	74 (24.5%)	78 (26.7%)	
School integration program			
* Yes*	64 (21.2%)	35 (12.0%)	0.003 *
* No*	238 (78.8%)	257 (88.0%)	
Place of residence			
* Urban*	292 (96.7%)	289 (99.0%)	0.057
* Rural*	10 (3.3%)	3 (1.0%)	
Nutritional status			
* Malnutrition*	1 (0.3%)	1 (0.3%)	0.170
* Risk of malnutrition*	5 (1.7%)	4 (1.4%)	
* Normal*	82 (27.2%)	80 (27.4%)	
* Overweight*	78 (25.8%)	102 (34.9%)	
* Obesity*	110 (36.4%)	89 (30.5%)	
* Severe obesity*	26 (8.6%)	16 (5.5%)	
Muscular strength			
* High*	57 (18.9%)	84 (28.8%)	0.005 *
* Low*	245 (81.1%)	208 (71.2%)	
Central obesity			
* Normal adiposity*	133 (44.0%)	139 (47.6%)	0.338
* Moderate adiposity*	45 (14.9%)	50 (17.1%)	
* Excess of adiposity*	124 (41.1%)	103 (35.3%)	

* *p* ≤ 0.05.

**Table 2:** Anthropometric characteristics of the total sample according to group and sex.

Total Sample	High Strength-NW *n* = 53	High Strength-OO *n* = 85	Low Strength-NW *n* = 109	Low Strength-OO *n* = 336	*p*-Values
Anthropometry					
*Age (years)*	11.9 [11.6–12.2]	11.9 [11.7–12.2]	12.3 [12.1–12.6]	12.0 [11.9–12.2]	0.094
*Weight (kg)*	44.3 [42.2–46.5] ^a^	60.7 [57.9–63.4] ^b^	45.0 [43.4–46.7] ^a^	61.4 [59.9–62.9] ^b^	0.000
*Height (m)*	1.53 [1.50–1.56]	1.54 [1.52–1.56]	1.52 [1.50–1.54]	1.52 [1.51–1.53]	0.470
*BMI (kg/m^2^)*	18.8 [18.4–19.2] ^a^	25.4 [24.6–26.1] ^b^	19.3 [19.0–19.6] ^a^	26.3 [25.8–26.7] ^b^	0.000
*WC (cm)*	65.49 [63.54–67.44] ^a^	75 [72.56–77.44] ^b^	65.61 [64.42–66.81] ^a^	78.11 [77.09–79.13] ^c^	0.000
*WtH (cm/cm)*	0.43 [0.42–0.44] ^a^	0.49 [0.47–0.50] ^b^	0.43 [0.43–0.44] ^a^	0.51 [0.51–0.52] ^c^	0.000
**Boys**	**High Strength-NW** ***n* = 23**	**High Strength-OO** ***n* = 33**	**Low Strength-NW** ***n* = 59**	**Low Strength-OO** ***n* = 181**	***p*-Values**
Anthropometry					
*Age (years)*	12.3 [11.9–12.8]	11.9 [11.4–12.3]	12.6 [12.2–12.9]	12.1 [11.9–12.2]	0.037
*Weight (kg)*	47.1 [44.1–50.1] ^a^	63.3 [57.4–69.3] ^b^	44.9 [42.5–47.4] ^a^	62.1 [60.2–64.1] ^b^	0.000
*Height (m)*	1.58 [1.53–1.62]	1.56 [1.52–1.60]	1.52 [1.49–1.55]	1.54 [1.52–1.56]	0.155
*BMI (kg/m^2^)*	18.8 [18.4–19.3] ^a^	25.7 [24.2–27.3] ^b^	19.2 [18.8–19.6] ^a^	26.0 [25.4–26.5] ^b^	0.000
*WC (cm)*	67.6 [64.1–71.1] ^a^	77.9 [73.4–82.5] ^b^	66.7 [64.9–68.5] ^a^	78.8 [77.3–80.3] ^b^	0.000
*WtH (cm/cm)*	0.43 [0.41–0.45] ^a^	0.50 [0.48–0.52] ^b^	0.44 [0.43–0.45] ^a^	0.51 [0.50–0.52] ^b^	0.000
**Girls**	**High Strength-NW** ***n* = 30**	**High Strength-OO** ***n* = 52**	**Low Strength-NW** ***n* = 50**	**Low Strength-OO** ***n* = 155**	***p*-Values**
Anthropometry					
*Age (years)*	11.6 [11.0–12.0]	12.0 [11.6–12.4]	12.1 [11.7–12.4]	12.0 [11.7–12.2]	0.403
*Weight (kg)*	42.2 [39.3–45.1] ^a^	59.0 [56.5–61.5] ^b^	45.2 [43.0–47.4] ^a^	60.5 [58.3–62.7] ^b^	0.000
*Height (m)*	1.49 [1.46–1.53]	1.53 [1.51–1.55]	1.52 [1.50–1.55]	1.50 [1.49–1.51]	0.099
*BMI (kg/m^2^)*	18.7 [18.1–19.4] ^a^	25.2 [24.4–26.0] ^b^	19.4 [18.9–19.9] ^a^	26.6 [25.9–27.3] ^b^	0.000
*WC (cm)*	63.9 [61.7–66.0] ^a^	73.1 [70.4–75.9] ^b^	64.4 [62.9–65.9] ^a^	77.3 [75.9–78.7] ^c^	0.000
*WtH (cm/cm)*	0.43 [0.42–0.44] ^a^	0.48 [0.46–0.50] ^b^	0.42 [0.41–0.43] ^a^	0.52 [0.51–0.53] ^c^	0.000

NW, normal weight; OO, overweight/obese; BMI: body mass index; WC, waist circumference; WtH, waist-to-height ratio. Statistical analysis was performed with one-way ANOVA. Average ^a, b, c^ within a row with a different symbol indicates significant differences between groups (one-way ANOVA and post hoc comparison with Bonferroni test). A *p* < 0.05 was considered for all analyses.

## Data Availability

Data available upon request due to ethical and privacy restrictions.
